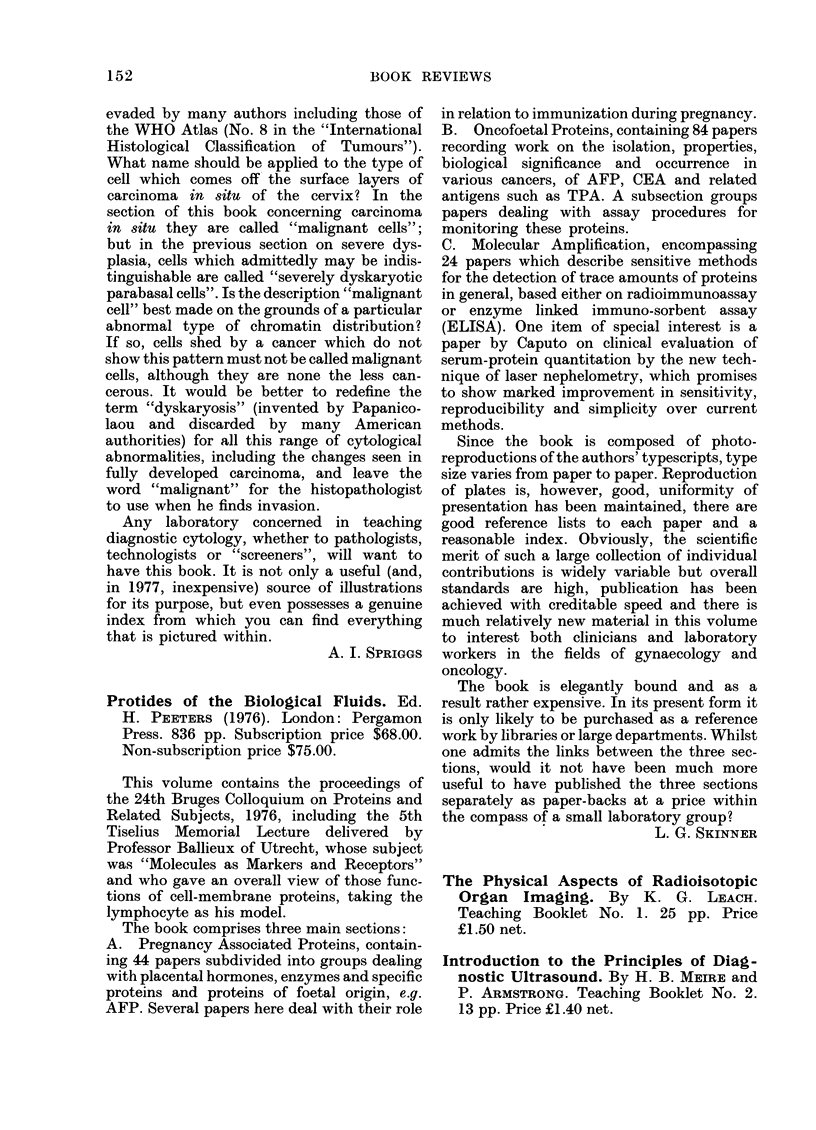# Protides of the Biological Fluids

**Published:** 1977-07

**Authors:** L. G. Skinner


**Protides of the Biological Fluids.** Ed. H. PEETERS (1976). London: Pergamon Press. 836 pp. Subscription price $68.00. Non-subscription price $75.00.

This volume contains the proceedings of the 24th Bruges Colloquium on Proteins and Related Subjects, 1976, including the 5th Tiselius Memorial Lecture delivered by Professor Ballieux of Utrecht, whose subject was "Molecules as Markers and Receptors" and who gave an overall view of those functions of cell-membrane proteins, taking the lymphocyte as his model.

The book comprises three main sections:

A. Pregnancy Associated Proteins, containing 44 papers subdivided into groups dealing with placental hormones, enzymes and specific proteins and proteins of foetal origin, *e.g.* AFP. Several papers here deal with their role in relation to immunization during pregnancy.

B. Oncofoetal Proteins, containing 84 papers recording work on the isolation, properties, biological significance and occurrence in various cancers, of AFP, CEA and related antigens such as TPA. A subsection groups papers dealing with assay procedures for monitoring these proteins.

C. Molecular Amplification, encompassing 24 papers which describe sensitive methods for the detection of trace amounts of proteins in general, based either on radioimmunoassay or enzyme linked immuno-sorbent assay (ELISA). One item of special interest is a paper by Caputo on clinical evaluation of serum-protein quantitation by the new technique of laser nephelometry, which promises to show marked improvement in sensitivity, reproducibility and simplicity over current methods.

Since the book is composed of photoreproductions of the authors' typescripts, type size varies from paper to paper. Reproduction of plates is, however, good, uniformity of presentation has been maintained, there are good reference lists to each paper and a reasonable index. Obviously, the scientific merit of such a large collection of individual contributions is widely variable but overall standards are high, publication has been achieved with creditable speed and there is much relatively new material in this volume to interest both clinicians and laboratory workers in the fields of gynaecology and oncology.

The book is elegantly bound and as a result rather expensive. In its present form it is only likely to be purchased as a reference work by libraries or large departments. Whilst one admits the links between the three sections, would it not have been much more useful to have published the three sections separately as paper-backs at a price within the compass of a small laboratory group?

L. G. SKINNER